# Timing of Home Health Care Initiation and 30-Day Rehospitalizations among Medicare Beneficiaries with Diabetes by Race and Ethnicity

**DOI:** 10.3390/ijerph18115623

**Published:** 2021-05-25

**Authors:** Jamie M. Smith, Haiqun Lin, Charlotte Thomas-Hawkins, Jennifer Tsui, Olga F. Jarrín

**Affiliations:** 1 College of Nursing, Thomas Jefferson University, Philadelphia, PA 19107, USA; jamie.smith3@jefferson.edu; 2 School of Nursing, Rutgers, The State University of New Jersey, Newark, NJ 07108, USA; haiqun.lin@rutgers.edu (H.L.); charlot@sn.rutgers.edu (C.T.-H.); 3 School of Public Health, Rutgers, The State University of New Jersey, Piscataway, NJ 08854, USA; 4 Keck School of Medicine of USC, University of Southern California, Los Angeles, CA 90033, USA; tsuijenn@usc.edu; 5 Institute for Health, Health Care Policy, and Aging Research, Rutgers, The State University of New Jersey, New Brunswick, NJ 08901, USA

**Keywords:** chronic conditions, diabetes, older adults, race or ethnicity, social determinants of health, inequalities or inequities, policy, health care access, home health care, rehospitalization

## Abstract

Older adults with diabetes are at elevated risk of complications following hospitalization. Home health care services mitigate the risk of adverse events and facilitate a safe transition home. In the United States, when home health care services are prescribed, federal guidelines require they begin within two days of hospital discharge. This study examined the association between timing of home health care initiation and 30-day rehospitalization outcomes in a cohort of 786,734 Medicare beneficiaries following a diabetes-related index hospitalization admission during 2015. Of these patients, 26.6% were discharged to home health care. To evaluate the association between timing of home health care initiation and 30-day rehospitalizations, multivariate logistic regression models including patient demographics, clinical and geographic variables, and neighborhood socioeconomic variables were used. Inverse probability-weighted propensity scores were incorporated into the analysis to account for potential confounding between the timing of home health care initiation and the outcome in the cohort. Compared to the patients who received home health care within the recommended first two days, the patients who received delayed services (3–7 days after discharge) had higher odds of rehospitalization (OR, 1.28; 95% CI, 1.25–1.32). Among the patients who received late services (8–14 days after discharge), the odds of rehospitalization were four times greater than among the patients receiving services within two days (OR, 4.12; 95% CI, 3.97–4.28). Timely initiation of home health care following diabetes-related hospitalizations is one strategy to improve outcomes.

## 1. Introduction

In the United States, nearly one in four older adults are living with diabetes, a condition associated with increased morbidity, mortality, and health care utilization [1,2,3]. In 2017, diabetes accounted for approximately 24% of inpatient spending and 21% of home health care spending in the United States [4]. Additionally, adults with diabetes experience higher rehospitalization rates [2] and have medical expenditures two times higher than those without diabetes [4]. For these patients, increased rehospitalization risk may be related to complicated transitions from hospital to home, complex medication regimens, and coexisting conditions that impact functional or cognitive status [5,6]. Effective post-acute home health care services can mitigate the risks of adverse events with prompt clinical assessment to address deterioration in condition and medication reconciliation [5,7,8]. Furthermore, skilled home health care services can support patients with diabetes by evaluating and reinforcing diabetes self-management skills, medication management, nutritional support, and glucose monitoring to prevent complications [9,10,11].

Facilitating a safe return home following hospitalization should be a collaboration between the patient, their families, and acute and outpatient providers. This involves discharge planning to identify patients who might benefit from post-acute home health care and coordinate the referral of services, as well as patient prioritization by home health agencies and prompt outpatient follow-up [12,13,14,15,16]. This collaborative process relies on numerous people, structures, and processes to create, communicate, and enact the discharge plan. Institutional and structural barriers can cause delayed or missed care. In recent studies of Medicare patients discharged to home health care in 2015–2016, 54% of all hospitalizations [17] and 73% of patients with a diabetes-related stay [18] received home health care within two weeks of discharge. Racial/ethnic disparities in post-acute referral and utilization of home health care were observed in both studies for non-Hispanic Black, Asian American/Pacific Islander (AAPI), American Indian/Alaska Native (AIAN), and Hispanic patients compared to (non-Hispanic) WHITE patients [17,18]. Efforts are ongoing to standardize institutional processes using clinical decision tools for referral decisions during the discharge planning and to prioritize home health visits at the agency level [12,19].

Preventing costly rehospitalizations and improving patient outcomes have been an ongoing focus of national policy and payment reforms. The Hospital Readmissions Reduction Program (HRRP) of the Centers for Medicare & Medicaid Services (CMS) incentivizes organizations to prioritize outcomes with the payment reform. In 2020, nearly half of all hospitals in the United States were financially penalized with lower Medicare reimbursements based on their higher than expected 30-day readmission rates between 2016–2019 [20]. Given the prevalence of diabetes, its contributions to other health conditions, and associated financial burden, addressing 30-day readmissions among patients with a diabetes-related hospitalization is important for patient outcomes and innovation in care delivery. One example of this effort is in the State Innovation Models (SIM) initiatives of the CMS, wherein improving diabetes care is a priority in every state program [21]. Building directly on our earlier paper [18] examining predictors of hospital discharge to home health care and post-acute home health care use among Medicare beneficiaries with diabetes, this paper explores the following question: what is the relationship between delayed, late, or missed home health care and 30-day all-cause rehospitalizations.

## 2. Materials and Methods

### 2.1. Study Design and Conceptual Framework

**Figure 1:**
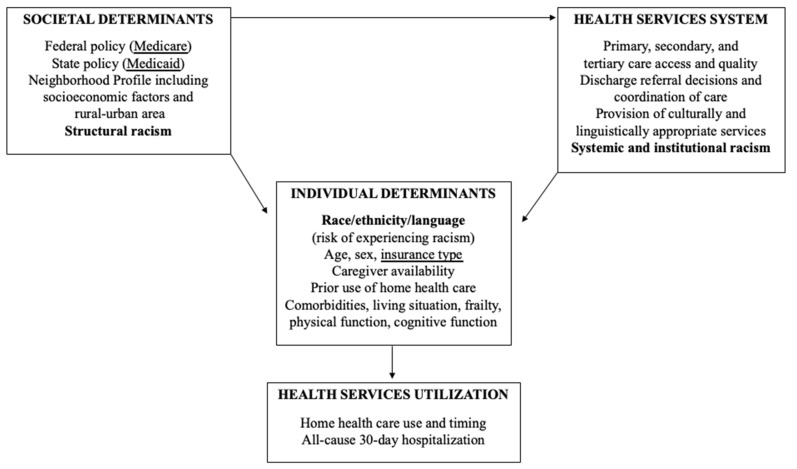
Conceptual model based on Andersen and Newman’s Framework for Viewing Health Services Utilization [18].

This was a retrospective analysis of Medicare fee-for-service and Medicare Advantage beneficiaries who experienced a diabetes-related hospitalization in 2015 that ended in discharge to home with a home health care referral or self-care [18]. Linked datasets utilized for this project include the Medicare Beneficiary Summary File (MBSF) (2014–2016, 100%), the inpatient Medicare Provider and Analysis Review (MedPAR) file, and the Home Health Outcome and Assessment Information Set (OASIS). The study design, selection of variables, and interpretation of results were guided by our adaptation [18] of Andersen and Newman’s Framework for Viewing Health Services Utilization (Figure 1) [22].

From this lens, societal determinants, including federal and state policy, neighborhood socioeconomic and geographic factors, and structural racism exert direct and indirect effects on the individual’s access to and utilization of primary care. Examples of health system resources and services that vary by geography encompass availability and type of primary care including home health care, secondary care including endocrinologists and insulin-pump providers, and tertiary care including potentially avoidable hospital stays.

### 2.2. Study Population

The study sample was constructed by identifying all unique, diabetes-related hospital admissions during 2015 among the national Medicare and Medicare Advantage population (100%) living within the United States (including Puerto Rico) (*n* = 1,270,929) [18]. We took into consideration the racial/ethnic, socioeconomic, and age disparities associated with diabetes onset, progression, and risk of serious complications (e.g., blindness, renal failure, infection, and amputation). We included Medicare beneficiaries aged 50 and older in our study population [18]. On average, non-Hispanic WHITE patients with diabetes are older than non-Hispanic Black, Asian, and Hispanic patients [23]. Diabetes-related hospitalizations were defined as either (1) a primary admitting diagnosis of diabetes or (2) a secondary diagnosis of diabetes and a diabetes-related condition including cardiovascular, renal, lower extremity, or eye diseases [24]. The list of International Classification of Diseases, ninth revision (ICD-9) and tenth revision (ICD-10) diagnosis codes used to identify diabetes-related hospitalizations in this study was previously reported [18]. The study population was restricted to patients continuously enrolled in Medicare for at least 12 months prior to the index hospitalization and were hospitalized during the 120 days prior to the index hospitalization [18]. Finally, we limited the sample to patients with a hospital discharge destination of home with home health care or home with self-care, resulting in a cohort of 786,734 Medicare beneficiaries [18].

### 2.3. Data Sources and Variables

The primary outcome was 30-day all-cause rehospitalization as identified from the Medicare Provider and Analysis Review File (MedPAR). The primary independent variable was the timing of post-acute home health care initiation, categorized as prompt (0–2 days), delayed (days 3–7), late (days 8–14), or not received. This variable was defined as the days from index hospital discharge (MedPAR) to the first post-acute home health assessment (OASIS). Hospital discharge destination (home to self-care or home with home health care) was extracted from the MedPAR file. Individual-level characteristics including age, sex, race/ethnicity, insurance, comorbidities, hospital length of stay, and use of home health care during the 120 days prior to the index hospitalization were extracted from the Medicare Beneficiary Summary File (MBSF) and OASIS files [18]. To minimize the frequency of unknown/other race and misclassification error, the imputed Research Triangle Institute (RTI) race variable contained in the Medicare Beneficiary Summary File (MBSF) was augmented with the patient’s self-reported race/ethnicity from the home health care assessment (OASIS) data [25,26]. We used six mutually exclusive racial/ethnic categories: non-Hispanic White, Black, Hispanic, Asian American/Pacific Islander (AAPI), American Indian/Alaska Native (AIAN), and unknown/other. Flags for end-stage renal disease and dementia included in the MBSF [18] supplemented the comorbidities from the Elixhauser Index calculated from the ICD-9 and ICD-10 codes present in the MedPAR file [27]. Geographic variables included the patient’s state of residence for which we used a dummy variable for each state to minimize the error associated with between-state variation in the Medicare Advantage and Medicaid programs.

The neighborhood profile variable was created by combining socioeconomic disadvantage and urban–rural classification into a four-category variable: (a) rural-advantaged, (b) rural-disadvantaged, (c) urban-advantaged, and (d) urban-disadvantaged [18]. Socioeconomic disadvantage was defined as living in a census tract classified at the 85th percentile or above on the 2015 Area Deprivation Index v2.0 composite of 17 socioeconomic indicators from the 2011–2015 U.S. Census American Community Survey [28]. ZIP codes were classified as rural or urban using the 2013 Economic Research Service’s Rural-Urban Continuum Codes (RUCC) for 5-digit ZIP codes [29]. Binary indicators for these two variables were linked to patients’ 9-digit ZIP codes using source data crosswalks [28,29].

### 2.4. Analytic Approach

In our cohort, home health care utilization and timing of services were influenced by individual patient needs, as well as by institutional and societal factors that impact discharge planning and availability of culturally and linguistically appropriate services. Historically, propensity score methods have been used to account for potential selection bias in observational studies and were first proposed by Rosenbaum and Rubin (1983) to balance the treatment groups on risk factors [30]. Constructing and incorporating a propensity score rather than adding additional risk factors directly to the outcome model has both conceptual and technical advantages. Conceptually, propensity scores can account for potentially confounding factors that may not be used to account for differences in the outcome such as neighborhood socioeconomic profile and unmeasured state differences in health policy. Technically, when there are large numbers of predictors, complex interactions and/or nonlinear relationships with the treatment groups may also be present that make them difficult to be directly included in the outcome model [31].

A multinomial logit model for the four categories of home health care timing was used to estimate the propensity scores. We included the variables associated with the timing of home health care and some interaction terms including prior home health care use, race/ethnicity (racism), insurance type, neighborhood profile, and selected comorbidities. Due to the high skewness of length of stay, we included the log transformation of it as a predictor in the propensity score model. Additional covariates used in the propensity score were hospital discharge destination, prior use of home health care, age group, sex, race/ethnicity, insurance type, state of residence, neighborhood socioeconomic profile, Elixhauser comorbidity index score, and comorbidities.

After propensity scores were estimated, patients were weighted by the inverse probability of them receiving the treatment they received based on the observed predictors in the analysis of outcomes including the stratified analyses based on race. To ensure the expected sample size equal to the original sample size, we used the stabilized weight of propensity score which has the proportion of the treatment in the entire cohort as the numerator and serves to numerically stabilize the weight in case the probability is small [32]. Inverse probability weighting approach uses all patients in the dataset and reweighs patients to increase (or decrease) the weights of those with probabilities lower (or greater) than expected under proportional assignment to the four home health care timing groups. The reweighted data set created a pseudo-population for which there is no confounding due to the included predictors, although unobserved confounding may still exist. The inverse probability weighting approach attempts to mimic a situation in which treatment is randomly allocated to individuals and is the most suitable one for our purpose. Inverse probability weighting estimation resulted in estimates that can be interpreted as the average treatment effect (ATE) for the entire cohort being studied.

All the analyses were performed using SAS statistical analysis software, version (9.4) (SAS Institute, Inc., Cary, NC, USA). The threshold for statistical significance was set at *p* < 0.05. These analyses are part of a larger study titled “Comparative Effectiveness of Home Care for Diverse Elders’ Outcomes” approved by the Institutional Review Board of Rutgers, The State University of New Jersey, and the privacy review board of the Centers for Medicare and Medicaid Services.

## 3. Results

### 3.1. Patient Characteristics by Timing of the First Home Health Care Visit

In our cohort (*n* = 786,734), 27.2% (213,766) of the patients received home health care within 14 days of hospital discharge [18], and 71.6% (153,132) of these patients received prompt services that started within two days of hospital discharge. The patients who received prompt and delayed services had a longer hospital length of stay than those patients who received late or no services. While the Elixhauser comorbidity index scores were similar, there were some differences in comorbidity diagnosis across the groups. The patients who received prompt and delayed services had higher rates of chronic pulmonary disease, congestive heart failure, complicated diabetes, and peripheral vascular diseases. A larger portion of the patients who received late care had end-stage renal disease. The patients with dementia received more prompt, delayed, and late home health care. A smaller portion of Hispanic, AIAN, and AAPI patients received prompt services. Greater proportions of fee-for-service/Medicaid beneficiaries received prompt, delayed, and late care. Among the patients who did not receive home health services, there were higher proportions of Medicare Advantage beneficiaries and the patients who were Hispanic, AIAN, or AAPI. Utilization of home health care during the 120 days prior to the index hospitalization was a predictor of receiving home health care after discharge, including among patients who were discharged home to self-care. Among the patients who had received home health care during the 120 days prior to the index hospital stay, nearly half (49%) received a home health care visit within two days of hospital discharge, and an additional 17% received a visit within two weeks of discharge.

The 30-day all-cause rehospitalization rate for the entire cohort (*n* = 122,743) was 15.6%. Among the patients who received home health care that started promptly (days 0–2 after discharge) or was delayed (days 3–8), 20% were rehospitalized (Table 1). In contrast, 40% of the patients were rehospitalized when services started late (days 8–14). Additional descriptive results are presented in Table 1 stratified by timing of home health care initiation.

### 3.2. Balance of Predictors after Propensity Score Weighting

The purpose of employing inverse probability-weighted treatment was to account for individual and societal characteristics that were related to home health care timing. We examined weight distribution across the patients to ensure balance and support validity of the weighting adjustment [31]. The stabilized weights of inverse propensity score have a minimum of 0.14 and a maximum of 10. No extremely large or small weights were present, supporting validity of the positivity assumption [31]. All the pairwise standardized mean differences after inverse probability weighting between the four home health care timings were within the recommended limits from −0.25 to 0.25 except for prior home health care use, indicating that all but one variable achieved satisfactory balance after inverse probability weighting. Even when a strict range such as from −0.1 to 0.1 was used, we still achieved satisfactory balance except for prior home health care use and discharge destination. To account for potential imbalance, prior home health care use and index hospitalization discharge destination were included in the final logistic models for the outcomes. For transparency, we presented the final logistic regression results predicting 30-day all-cause rehospitalization with and without inverse propensity score weighting in Table 2. The results were similar, with slightly smaller effects of home health care timing in the unweighted models.

### 3.3. Home Health Care Timing and Rehospitalization

Table 2 presents the results of the logistic regression models predicting 30-day all-cause rehospitalization in the full cohort stratified by hospital discharge destination. In the full cohort, home health care initiated after two days was associated with higher odds of rehospitalization. When the results were stratified by hospital discharge destination, two distinct patterns were observed (Table 2). Among the patients discharged home to self-care, initiation of home health care on day 3–7 was associated with 39% higher odds of 30-day rehospitalization compared to the initiation of services within two days (*p* < 0.001). However, among the patients discharged to home health care, there was no significant difference in rehospitalization risk between the patients who received home health care within two days compared to later in the first week. In contrast, when home health care was initiated more than a week after hospital discharge (on post-acute day 8–14), the odds of rehospitalization were dramatically higher among the patients discharged to self-care (OR, 4.71; 95% CI, 4.51–4.94) as well as among the patients discharged to home health care (OR, 2.53; 95% CI, 2.36–2.72). Finally, among the patients discharged to home health care who did not receive services within 14 days, the odds of rehospitalization were higher (OR, 1.18; 95% CI, 1.15–1.21) compared to their counterparts who received home health care services within seven days (*p* < 0.001).

Across all the racial/ethnic groups, the overall results mirrored those above, i.e., the patients receiving delayed or late home health care were significantly more likely to be rehospitalized compared to the patients receiving prompt home health care (Table 3). In the overall models stratified by race/ethnicity, the relationship between the timing of home health care initiation and rehospitalization was significant across all racial/ethnic groups; however, the results differed when further stratified by discharge destination. Among the patients discharged to home health who did not receive services, odds of rehospitalization were higher compared to the patients who received an initial visit within two days for White (OR, 1.20; 95% CI, 1.16–1.24) and Black (OR, 1.21; 95% CI, 1.13–1.29) patients, but not for other racial/ethnic groups. Table 3 presents additional logistic regression results stratified by discharge destination.

## 4. Discussion

This study explored the relationship between the timing of home health care initiation and 30-day rehospitalization risk among Medicare and Medicare Advantage beneficiaries following a diabetes-related hospitalization. In our study population, we found that when home health care was delayed after hospital discharge, the patients were more likely to experience a 30-day rehospitalization. Our findings support the standard of care set by the Centers for Medicare and Medicaid Services that skilled home health care services should begin within 48 h of referral or hospital discharge (if later) unless the physician/provider authorizes a delay in initiation of services due to an outpatient visit or a request of the patient or family [33]. After accounting for physician/provider authorized exceptions for outpatient visits or patient/family preference, over 95% of home health care patients in the United States receive care that begins within two days of referral or hospital discharge [34]. In comparison, within our cohort, only 72% of patients (153,132/213,766) who received home health care had services initiated within two days of hospital discharge. This difference may be partially explained by situations where a patient was discharged to self-care and a need for home health care may have been identified at a follow-up appointment. Additionally, planned use of home health care may have been approved by the patient’s physician (and thus counted as an exception to the two-day rule) to not conflict with an outpatient medical appointment, dialysis session, or preference of the patient/family. In other studies, the combination of early home health care and outpatient provider follow-up has been associated with a reduced readmission rate in patients with heart failure [15] and sepsis [35]. Future analysis with outpatient claims data would be helpful in describing the relationship between home health care timing, outpatient visits, and rehospitalization in patients with diabetes.

In our study population, non-Hispanic White and Black patients who were discharged to home health care but did not receive services within seven days had significantly greater odds of rehospitalization compared to those who did (Table 3). This is particularly significant given a recent study finding no evidence that the recent SIM initiative of the CMS was effective in reducing readmission rates among adults with diabetes [21]. Our study’s findings suggest that prompt home health care initiation mitigates rehospitalization risks in these groups, supporting the benefit of timely post-acute care at home.

While identifying the causes for rehospitalization was beyond the scope of this study, other work has identified factors such as requiring assistance with medication regimens and management of other chronic illnesses as potential risks for readmission among patients with diabetes [6,36]. A possible explanation for the reduced rehospitalization rate with early home health care found in this study could be specific to the patient’s needs following diabetes-related hospitalization. For example, escalation in medication treatment during a hospital stay, such as beginning insulin therapy, which has been identified as a significant predictor of 30-day readmission in a sample of patients with diabetes [37]. Post-acute home health care reduces the risk of adverse events through medication reconciliation; patient and caregiver education; coordinating and scheduling follow-up care; transportation; and deliveries of supplies and social services if needed [6,7].

Diabetes disproportionally burdens racial/ethnic minority groups [38,39,40,41]. Prior research found home health care services were underutilized by AAPI patients [42,43]. We are unaware of any literature describing home health care use and outcomes among AIAN patients who, despite having a high prevalence of diabetes [44], are infrequently included in research due to relatively small numbers. In this study, AIAN and AAPI patients who were discharged to home health care were 20% less likely to receive services compared to their WHITE counterparts [18]. In the results presented in this paper (Table 3), AAPI and AIAN patients were at the greatest overall risk of rehospitalization compared to other racial and ethnic groups when home health care services were received more than two days after hospital discharge in weighted models. These results should be interpreted with caution due to weighting of small numbers (AAPI, *n* = 19,888; AIAN, *n* = 5859) but warrant further investigation to better understand these findings.

Timing of the initial home health visit is not the sole determinant of rehospitalization risk. Structural determinants and institutional factors may contribute to differences in the effects of home health care timing on rehospitalization risk across racial/ethnic groups. Although we accounted for neighborhood socioeconomic advantage, other unmeasured societal determinants may contribute to rehospitalization risk. Racial residential segregation [45] and residence in food swamps [46] contribute to increased rehospitalization risk among patients with diabetes. These societal determinants are examples of structural racism embedded in the community’s infrastructure, compounding the impact of unequal health care resource distribution/access [47,48,49]. Communities with concentrated poverty, higher crime rates, and ethnic enclaves requiring utilization of interpreter services may directly or indirectly contribute to a home health agency’s ability to provide timely care. For example, when home health agency nurses require security escorts or interpreter services, there is no adjustment for this in the payment provided by Medicare and Medicaid, leaving these costs to be absorbed by the agency. Administrative challenges with managed care plans, such as complicated authorization processes that delay start of care, lower payment rates, increase administrative costs and may create barriers to timely post-acute care [50]. The recent addition of social determinants of health ICD-10-CM codes for socioeconomic and psychosocial circumstances [51] provides one potential mechanism to adjust reimbursement rates to home health agencies while maintaining expectations for prompt initiation of home health care and patient outcomes.

When stratified by hospital discharge destination and race/ethnicity, the study’s findings support the need for evidence-based processes and highlight areas for potential future work. For example, approximately 28% of the study patients who received home health care had been discharged to self-care [18] since the rehospitalization risk with late home care initiation was greater within this group; further work is needed to understand why patients were not referred to services during discharge planning. While efforts to standardize discharge planning and visit prioritization are ongoing, most decisions are still subjective and rely on a provider’s decision-making and communication [12,19]. Qualitative work has suggested the contribution of community demographics and lack of workforce diversity affect home health care services for racial/ethnically diverse patients [52]. In a study of discharge planners, time constraints and insurance concerns were reported as barriers contributing to a 20% difference in referrals between WHITE and Hispanic patients [53]. Additionally, it is important to consider how the patients’ prior experiences with inpatient and community care may positively or negatively impact acceptance of a discharge plan that includes home health care [54,55]. This is especially important among patients who are not referred to home health care at hospital discharge but are eligible for services, highlighting the importance of early identification of patients that may benefit from home health care prior to hospital discharge. Health care organizations must engage with the communities they serve and collaborate with them on interventions that can best improve equitable care delivery [56]. Further, they must assess institutional policies and practices to assess for biases and audit compliance with the National Standards for Culturally and Linguistically Appropriate Services [56,57]. Future research should seek to understand the discharge and transitional care process from the perspective of racial/ethnic minority patients and families to better understand the factors contributing to delays in receiving home health care services [58].

The study had several limitations. First, we do not know the reasons for delayed, late, and missed care. Factors such as patient’s preferences, home environment, marital status, or caregiver availability have been associated with home health utilization [42,43,55] but were not contained in hospital discharge records available at the population level for Medicare beneficiaries. Second, we did not utilize outpatient claims data and thus did not account for the possibility that outpatient follow-up visits contributed either to reducing the risk of rehospitalization or to delaying home health initiation [15,36,59]. Third, there may be unmeasured individual, health care system, or geographic factors that we did not include in the models. Finally, we did not account for differences between Medicare Advantage plans, some of which have copays or more extensive prior authorization procedures associated with reduced utilization of home health care services [60], and potential delays to start of care [50].

## 5. Conclusions

This paper provides evidence for the value of home health care services as a strategy to reduce the 30-day hospital readmission rate among adult Medicare beneficiaries with diabetes. Our overall finding that timely initiation of home health care was associated with lower risks of 30-day rehospitalization supports the CMS requirement that home health care services be required within two days of hospital discharge when ordered, with the exception when the physician/provider authorizes a delay in the initiation of services due to an outpatient visit or the patient’s or family’s request. Furthermore, the patients who benefited the most from receiving home health care services within two days of discharge were those who were at risk of falling between the cracks, who were discharged home to self-care—yet received a timely home health care visit anyway. These findings support the health care providers’ and discharge planners’ efforts to identify patients with a diabetes-related hospitalization who may need home health services and whose discharge plan and referral may require extra time, including patients who have recently utilized home health care or who may have cost-sharing or prior authorization requirements [60].

## Figures and Tables

**Table 1:** Sample characteristics and outcome by timing of the first home health care visit, row percentage displayed.

		Timing of the First Home Health Care Visit			
	Total *n* = 786,734	Day 0–2 153,132 (19.5)	Day 3–7 46,659 (5.9)	Day 8–14 13,975 (1.8)	Not Received 572,968 (72.8)
Race/Ethnicity					
White	534,725	108,661 (20.3)	29,450 (5.5)	8806 (1.6)	387,808 (72.5)
Black	134,246	26,321 (19.6)	10,443 (7.8)	3045 (2.3)	94,437 (70.3)
Hispanic	86,824	13,897 (16.0)	5233 (6.0)	1665 (1.9)	66,029 (76.0)
Asian American/Pacific Islander	19,888	3409 (17.1)	1234 (6.2)	365 (1.8)	14,880 (74.8)
American Indian/Alaska Native	5859	834 (14.2)	299 (5.1)	94 (1.6)	4632 (79.1)
Unknown	5192	-	-	-	5192 (100)
Sex, male	402,779	70,416 (17.5)	20,060 (5.0)	6283 (1.6)	306,020 (76.0)
Sex, female	383,955	82,716 (21.5)	26,599 (6.9)	7692 (2.0)	266,948 (69.5)
Age (x, SD)	73.1 (9.7)	75.50 (10.1)	75.14 (10.1)	74.54 (10.2)	72.30 (9.4)
Insurance					
Fee-for-service (FFS)	363,675	70,665 (19.4)	19,692 (5.4)	5882 (1.6)	267,436 (73.6)
FFS + Medicaid	143,162	36,078 (25.2)	10,664 (7.4)	3312 (2.3)	93,108 (65.0)
Medicare Advantage (MA)	189,393	29,665 (15.7)	10,289 (5.4)	2923 (1.5)	146,516 (77.4)
MA + Medicaid	90,504	16,724 (18.5)	6014 (6.6)	1858 (2.1)	65,908 (72.8)
Neighborhood profile					
Urban, advantaged	549,157	107,109 (19.5)	32,822 (6.0)	9558 (1.7)	399,668 (72.8)
Urban, disadvantaged	98,567	19,162 (19.4)	6777 (6.9)	2057 (2.1)	70,571 (71.6)
Rural, advantaged	106,827	20,191 (18.9)	5144 (4.8)	1682 (1.6)	79,810 (74.7)
Rural, disadvantaged	32,183	6670 (20.7)	1916 (6.0)	678 (2.1)	22,919 (71.2)
Elixhauser CI (x, SD)	30.0 (16.5)	31.61 (16.5)	32.22 (16.6)	31.93 (16.6)	26.53 (16.3)
Common comorbidities					
Chronic pulmonary disease	206,479	45,509 (22.0)	13,890 (6.7)	4117 (2.0)	142,963 (69.2)
Congestive heart failure	294,105	68,420 (23.2)	20,965 (7.1)	6011 (2.0)	198,709 (67.6)
Dementia	128,668	39,060 (30.4)	12,055 (9.4)	3507 (2.7)	74,046 (57.5)
Depression	89,824	19,688 (21.9)	6188 (6.9)	1726 (1.9)	62,222 (69.3)
Diabetes, complicated	231,299	52,197 (22.6)	15,738 (6.8)	4626 (2.0)	158,738 (68.6)
End-stage renal disease	62,900	10,960 (17.4)	4609 (7.3)	1468 (2.3)	45,863 (72.9)
Fluid/electrolyte	273,619	60,449 (22.1)	18,910 (6.9)	5518 (2.0)	188,742 (69.0)
Hypertension	706,560	136,437 (19.3)	41,964 (5.9)	12,549 (1.8)	515,610 (73.0)
Peripheral vascular disease	135,577	29,913 (22.1)	8559 (6.3)	2652 (2.0)	94,453 (69.7)
Prior home health care (120 days)	120,823	58,908 (48.8)	16,149 (13.4)	3866 (3.2)	41,900 (34.7)
Length of stay in days (x, SD)	3.9 (3.4)	5.2 (4.1)	4.6 (4.2)	4.3 (3.6)	3.4 (3.0)
Discharged to home health care	209,150	120,193 (57.5)	27,979 (13.4)	4029 (1.9)	56,949 (27.2)
Discharged to home with self-care	577,584	32,939 (5.7)	18,680 (3.2)	9946 (1.7)	516,019 (89.3)
Rehospitalization within 30 days (outcome)	122,740	30,126 (24.5)	9314 (7.6)	5626 (4.6)	77,674 (63.3)

Note: Not received = no evidence of home health care starting within 14 days of the index hospitalization; Elixhauser CI = Elixhauser comorbidity index score with hospital readmission weights.

**Table 2:** Results of weighted and unweighted logistic regression predicting 30-day all-cause rehospitalization stratified by discharge destination.

Home Health Care Timing	Overall	Discharged to Self-Care	Discharged to Home Health Care
Full Cohort Reference = day 0–2	OR, 95% CI	OR, 95% CI	OR, 95% CI
With propensity score weighting			
Delayed (day 3–7)	1.28, 1.25–1.32 ***	1.39, 1.34–1.43 ***	1.00, 0.95–1.06
Late (day 8–14)	4.12, 3.97–4.28 ***	4.72, 4.52–4.94 ***	2.53, 2.36–2.72 ***
No home health care received	0.98, 0.97–1.00 ***	0.85, 0.84–0.87 ***	1.18, 1.15–1.21 ***
Without propensity score weighting			
Delayed (day 3–7)	1.08, 1.05–1.11 ***	1.18, 1.12–1.23 ***	0.99, 0.96–1.02
Late (day 8–14)	3.28, 3.16–3.41 ***	3.42, 3.26–3.60 ***	2.43, 2.27–2.60 ***
No home health care received	0.96, 0.95–0.98 ***	0.85, 0.82–0.88 ***	1.15, 1.12–1.18 ***

Note: *** *p* < 0.001.

**Table 3:** Results of weighted logistic regression predicting 30-day all-cause rehospitalization stratified by race/ethnicity and discharge destination.

Home Health Care Timing	Overall	Discharged to Self-Care	Discharged to Home Health Care
Reference = day 0–2	OR, 95% CI	OR, 95% CI	OR, 95% CI
White	*n* = 534,725	*n* = 390,464	*n* = 144,261
Delayed (day 3–7)	1.33, 1.29–1.38 ***	1.44, 1.38–1.50 ***	1.04, 0.98–1.11
Late (day 8–14)	4.54, 4.34–4.75 ***	5.16, 4.89–5.44 ***	2.76, 2.53–3.01 ***
No home health care received	0.98, 0.96–1.00	0.83, 0.81–0.85 ***	1.20, 1.16–1.24 ***
Black	*n* = 134,246	*n* = 96,164	*n* = 38,082
Delayed (day 3–7)	1.16, 1.08–1.24 ***	1.27, 1.17–1.38 ***	0.94, 0.83–1.06
Late (day 8–14)	3.32, 3.03–3.64 ***	3.93, 3.52–4.39 ***	2.11, 1.78–2.52 ***
No home health care received	1.00, 0.96–1.04	0.89, 0.84–0.93 ***	1.21, 1.13–1.29 ***
Hispanic	*n* = 86,824	*n* = 66,989	*n* = 19,835
Delayed (day 3-7)	1.11, 1.02–1.21 *	1.18, 1.07–1.31 ***	0.89, 0.75–1.06
Late (day 8–14)	3.04, 2.70–3.41 ***	3.30, 2.89–3.77 ***	2.20, 1.73–2.80 ***
No home health care received	0.94, 0.90–0.99 *	0.88, 0.83–0.93 ***	1.07, 0.97–1.17
Asian American/Pacific Islander	*n* = 19,888	*n* = 14,590	*n* = 5298
Delayed (day 3–7)	1.52, 1.27–1.83 ***	1.94, 1.56–2.42 ***	0.93, 0.66–1.31
Late (day 8–14)	4.72, 3.69–6.03 ***	6.88, 5.14–9.21 ***	1.91, 1.16–3.14 *
No home health care received	1.09, 0.98–1.22	1.19, 1.03–1.37	0.93, 0.78–1.12
American Indian/Alaska Native	*n* = 5859	*n* = 4682	*n* = 1177
Delayed (day 3–7)	1.50, 1.08–2.09, *p* < 0.05	1.83, 1.24–2.70 **	0.49, 0.21–1.16
Late (day 8–14)	4.69, 2.95–7.44 ***	5.97, 3.48–10.25 ***	1.35, 0.40–4.54
No home health care received	0.94, 0.77–1.16	0.74, 0.57–0.96	1.52, 0.99–2.33

Notes: * *p* < 0.05, ** *p* < 0.01, *** *p* < 0.001.

## Data Availability

Restrictions apply to the availability of these data. The data were obtained from the CMS/ResDAC and are available from the authors with the permission of the CMS/ResDAC.
